# Enhanced C/EBPα Function Extends Healthspan and Lifespan in the African Turquoise Killifish

**DOI:** 10.1111/acel.70211

**Published:** 2025-09-19

**Authors:** Christine Müller, Joscha S. Muck, Kirill Ustyantsev, Gertrud Kortman, Josephine Hartung, Eugene Berezikov, Cornelis F. Calkhoven

**Affiliations:** ^1^ European Research Institute for the Biology of Ageing (ERIBA), University Medical Center Groningen (UMCG) University of Groningen Groningen the Netherlands

**Keywords:** C/EBP, healthspan, killifish, lifespan, translation

## Abstract

The transcription factor CCAAT/enhancer binding protein alpha (C/EBPα) regulates cell differentiation, proliferation, and function in various tissues, including the liver, adipose tissue, skin, lung, and hematopoietic system. Studies in rats, mice, humans, and chickens have shown that CEBPA mRNA undergoes alternative translation initiation, producing three C/EBPα isoforms. Two of these isoforms act as full‐length transcription factors with N‐terminal transactivation domains and a C‐terminal dimerization and DNA‐binding domain. The third isoform is an N‐terminally truncated variant, translated from a downstream AUG codon. It competes with full‐length isoforms for DNA binding, thereby antagonizing their activity. Expression of the truncated C/EBPα isoform depends on the initial translation of a short upstream open reading frame (uORF) in the CEBPA mRNA and subsequent re‐initiation at a downstream AUG codon, a process stimulated by mTORC1 signaling. We investigated whether the ortholog of the CEBPA gene in the evolutionarily distant, short‐lived African turquoise killifish ( *Nothobranchius furzeri* ) is regulated by similar mechanisms. Our findings reveal that the uORF‐mediated regulation of C/EBPα isoform expression is conserved in killifish. Disruption of the uORF selectively eliminates the truncated isoform, leading to unrestrained activity of the full‐length C/EBPα isoforms. This genetic modification significantly extended both the median and maximum lifespan and improved the healthspan of male *N. furzeri* . Furthermore, comparative transcriptome analysis revealed an upregulation of genes and pathways that are associated with healthspan and lifespan regulation in other species. These results highlight a conserved mechanism of *CEBPA* gene regulation across species and its potential role in modulating the lifespan and aging phenotypes.

## Graphical abstract

Mutation of the upstream open reading frame (uORF) in the *CEBPA* gene in short‐lived killifish, resulting in unrestrained activity of the C/EBPα transcription factor, is associated with increased healthspan and lifespan.

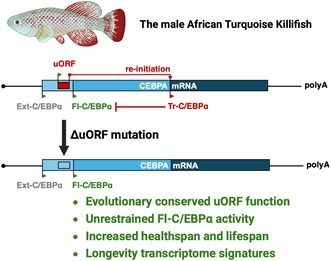

## 1 Introduction

The African turquoise killifish, *Nothobranchius furzeri*, is a unique vertebrate species that has attracted considerable attention in the field of aging research because of its exceptionally short lifespan. Native to ephemeral ponds in the semi‐arid regions of Mozambique and Zimbabwe, this species has evolved to complete its life cycle rapidly, often within a few months, to adapt to the transient availability of water in its natural habitat. These killifish show a pronounced and well‐characterized aging process in captivity, displaying many of the hallmarks of aging observed in longer‐lived vertebrates, including declines in cognitive and physical performance, changes in tissue integrity, increases in cancer incidence, and alterations in gene expression profiles (Genade et al. 2005; Hu and Brunet 2018; Reichard et al. 2015). *N. furzeri* has a fully sequenced genome, and genetic tools have been developed, including CRISPR/Cas9‐mediated gene editing (Bedbrook et al. 2023; Cui et al. 2020; Harel et al. 2016; Petzold et al. 2013; Reichwald et al. 2015) (see for genome browser https://nfingb.leibniz‐fli.de).

The CCAAT/enhancer binding protein family of basic region leucine‐zipper (bZIP) transcription factors consists of six members (designated α to ζ) that are widely expressed and involved in cell proliferation, differentiation, metabolism, and senescence (Lopes‐Paciencia et al. 2019; Nerlov 2007; Ramji and Foka 2002). C/EBPs regulate gene transcription by forming dimers and binding to C/EBP‐specific recognition sites in the genome. The expression of C/EBPα and C/EBPβ proteins is uniquely regulated at the level of mRNA translation, involving a *cis*‐regulatory upstream open reading frame (uORF) and translation into three protein isoforms of different lengths from a single mRNA molecule (Calkhoven et al. 1994, 2000). From long to short, C/EBPα comprises extended‐, full‐length(p42)‐, and truncated(p30)‐C/EBPα isoforms. The extended‐C/EBPα is translated from an unconventional CUG codon in most vertebrates or a GUG codon in humans. Its expression is generally low, and it plays a more specialized role in activating Polymerase I‐controlled ribosomal DNA transcription and the regulation of cell size (Muller et al. 2010). The p42 isoform is translated from the first AUG codon in the *CEBPA* reading frame and functions as a full transactivating factor. It contains an N‐terminal transactivation domain and protein–protein interaction domains, as well as a C‐terminal dimerization and DNA‐binding domain. P42‐C/EBPα is involved in activating Polymerase II‐mediated transcription of specific target genes. The third p30‐C/EBPα isoform is translated from a downstream in‐frame AUG codon, which depends on the preceding translation of the uORF and the subsequent re‐initiation at the downstream AUG codon. This isoform lacks most of the N‐terminal domains for transactivation but retains the C‐terminal dimerization and DNA‐binding domains. By competing with p42‐C/EBPα for DNA binding, it acts as a competitive inhibitor for p42‐C/EBPα. Mutation of the uORF abolishes p30‐C/EBPα expression, relieving its inhibitory action on p42‐C/EBPα and resulting in the unrestrained activity of p42‐C/EBPα (Calkhoven et al. 1994; Ossipow et al. 1993).

The present study was preceded by investigations of the regulation and function of C/EBPβ isoforms (Calkhoven et al. 2000; Zidek et al. 2015). These studies showed that mutation of the uORF (ΔuORF) in the *CEBPB* gene in cell lines or mice similarly abolishes the expression of the truncated C/EBPβ isoform called C/EBPβ‐LIP, thereby unleashing the transactivating function of the full‐length C/EBPβ‐LAP isoform, resulting in C/EBPβ superfunction. C/EBPβ‐ΔuORF mutation in mice induces calorie‐restriction type metabolic phenotypes, reduces spontaneous cancer incidence, and extends the median (20%) and maximum (10%) lifespan, the latter only in females (Muller et al. 2018, 2022; Zidek et al. 2015).

Here, we investigated whether different *N. furzeri* (Nf)C/EBPα isoforms are expressed in killifish and whether the truncated NfC/EBPα isoform depends on uORF translation, as observed in higher vertebrates. Our findings demonstrate that uORF‐mediated regulation of NfC/EBPα isoforms is conserved in *N. furzeri* . Disruption of uORF function selectively abolishes the truncated NfC/EBPα isoform, leading to enhanced transcriptional activity of the full‐length isoform. In vivo, this genetic modification significantly extends both median and maximum lifespan while improving certain healthspan parameters in males, which is correlated with changes in the transcriptome associated with health‐ and lifespan regulation. In contrast to males, females showed no changes in healthspan or lifespan, indicating a sex‐specific response to uORF‐dependent regulation of NfC/EBPα proteins in *N. furzeri* .

## 2 Results

### 2.1 CEBPA mRNA and C/EBPα Protein Structures Are Conserved in Vertebrates

**FIGURE 1:**
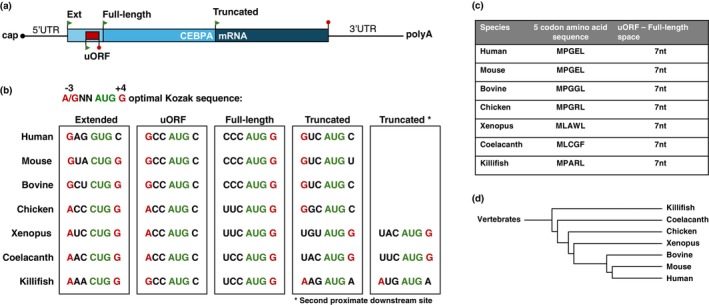
Evolutionary conservation of the CEBPA mRNA primary structure. (a) Schematic representation of CEBPA mRNA with 5′cap, 5′ and 3′ untranslated regions (UTR), translation initiation sites (green arrow points) for extended (Ext), full‐length and truncated C/EBPα protein isoforms, the upstream open reading frame (uORF), and polyadenylation tail (polyA). The uORF is +2 nt out of frame with the CEBPA coding frame. (b) Comparison of CEBPA translation initiation sites in different species and their adherence to the Kozak consensus sequence for optimal translation initiation shown above. (c) Pentapeptide sequences of uORFs from different species and the space between the uORF stop codon and the initiation codon for full‐length C/EBPα in nucleotides (nt). (d) Phylogenetic tree of the depicted species (generated with https://phylot.biobyte.de).

The conservation of the mRNA primary structure throughout vertebrate evolution is remarkable, as demonstrated by the alignment of mRNA sequences of killifish with those from human, mouse, chicken, Xenopus, and the ancient coelacanth (Figure 1a–d). It encompasses the precise position of the uORF, distribution of translation initiation sites, and their relative strength, as determined by their adherence to the Kozak consensus sequence (Kozak 1984). This conservation predicts the expression of both extended and full‐length isoforms as well as the uORF‐dependent translation of a truncated isoform through mechanisms similar to those described for orthologous transcripts in other vertebrates (Calkhoven et al. 2000).

**FIGURE 2:**
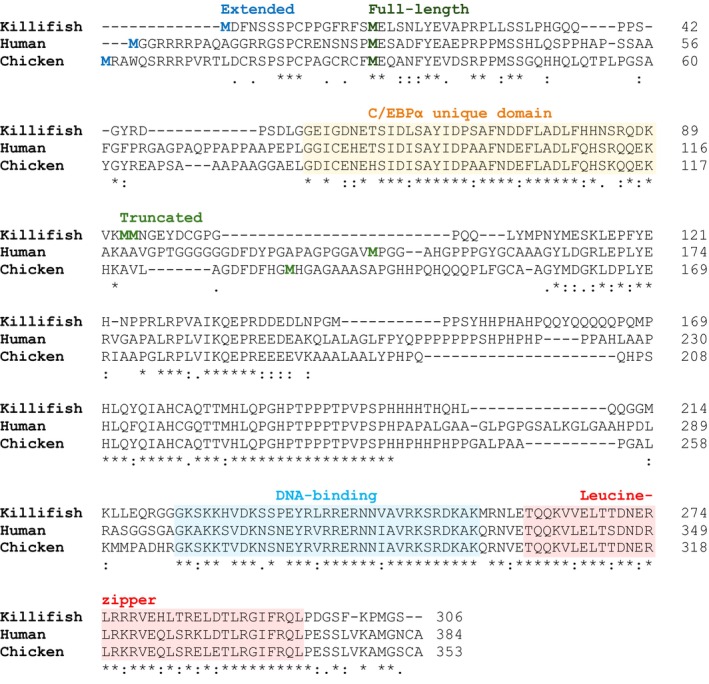
C/EBPα sequence alignment. Amino acids are aligned and marked conserved (*), conservative changes (:), and semi‐conservative changes (.). The initiating methionines for the Extended isoform are highlighted in blue, corresponding to the alternative start codons CUG (in killifish and chicken) or GUG (in humans). The initiating methionines for Full‐length and Truncated isoforms are highlighted in dark and light green, respectively. The unique N‐terminal transactivation domain of C/EBPα is marked yellow, and the C‐terminal conserved DNA‐binding and leucine‐zipper dimerization domains (bZIP) are marked in blue and red, respectively.

The killifish genome contains a single copy of the *NfCEBPA* gene ( *N. furzeri* Network genome (https://nfingb.leibniz‐fli.de) and transcriptome (https://nfintb.leibniz‐fli.de/nfintb/) browsers). Alignment of the NfC/EBPα protein sequence, derived from the genomic transcript of the *NfCEBPA* gene, with C/EBPα protein sequences from human, and chicken revealed strong conservation in the carboxy‐terminal region. This region contains a basic DNA‐binding domain and a leucine zipper dimerization domain (bZIP domain) (Figure 2) (Gene ID: 107381504; Petzold et al. 2013). The amino‐terminal region also contains conserved sequences associated with transactivation, specifically the domain spanning amino acids 36 and 73 in the killifish sequence. This domain is unique to C/EBPα and absent in other C/EBP family members, confirming the identity of the sequence as C/EBPα. Two methionine residues, located downstream of the transactivation domain, are predicted to serve as initiation sites for the truncated isoform. Additionally, an upstream CUG codon in a favorable Kozak context is predicted to function as an initiation site for an N‐terminally extended isoform, with similar initiation codons observed across species (Figure 1b).

### 2.2 uORF‐Dependent Translation of the NfCEBPA mRNA Into Three Protein Isoforms

**FIGURE 3:**
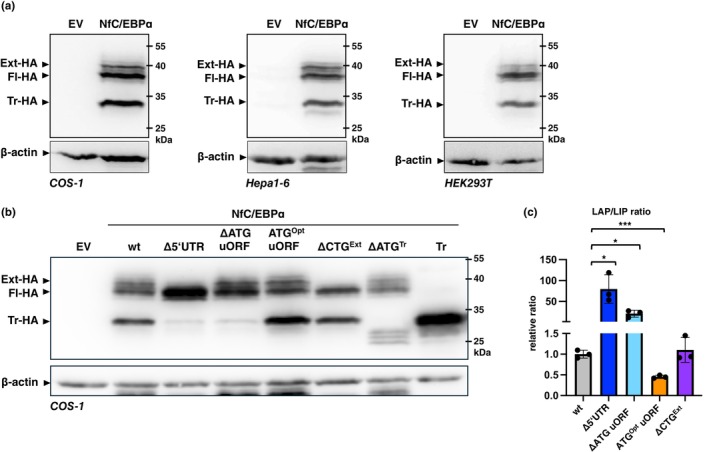
Differential translation of CEBPA mRNA into three NfC/EBPα protein isoforms. (a) COS‐1, Hepa1‐6, and HEK293 cells were transfected with the wild‐type (wt) NfC/EBPα‐HA expression vector, and protein expression was determined by immunoblotting using anti‐HA antibodies. The NfC/EBPα‐HA protein isoforms are labeled as extended (Ext‐HA), full‐length (Fl‐HA), and truncated (Tr‐HA). β‐Actin served as a loading control. (b) COS‐1 cells were transfected with NfC/EBPα‐HA expression vectors containing the following mutations: Deletion of the entire 5′UTR (Δ5′UTR), deletion of the uORF‐ATG (ΔATG), placing the uORF‐ATG in a Kozak sequence for optimal translation initiation efficiency (ATG^Opt^), deletion of the predicted extended‐CUG initiation codon (ΔCTG^Ext^), deletion of the double AUG predicted as an initiation site for the truncated isoform (ΔATG^Tr^), and an expression vector for truncated (Tr)‐NfC/EBPα only. β‐Actin served as a loading control. (c) Bar graph showing quantifications of the relative changes in LAP/LIP isoform ratios by chemiluminescence digital imaging from three biological replicates (panel b and Figure S1a). Values are mean ± SD; significance was determined by Student's *t*‐tests, *p < 0.05, ***p < 0.001.

To analyze NfC/EBPα protein isoform expression, we cloned the *NfCEBPA* cDNA sequence into the eukaryotic expression vectors pCDNA3.1 and pSG5. Owing to the lack of anti‐NfC/EBPα antibodies, a hemagglutinin (HA) epitope was added at the C‐terminus. Transfection of the wild‐type (wt) cDNA in COS‐1, HEK293, and Hepa1‐6 cell lines resulted in the expression of the three expected protein isoforms, labeled extended (Ext), full‐length (Fl), and truncated (Tr) (Figure 3a). Next, we examined the usage of the predicted translation initiation sites and assessed the consequences of their perturbation on the expression of NfC/EBPα isoform in COS‐1 cells (Figure 3b,c, and Figure S1a). Removal of the entire 5′UTR (Δ5′UTR) led to the exclusive expression of Fl‐NfC/EBPα, indicating that the regulatory sequences required for additional isoform expression reside within the 5′UTR. Mutation of uORF‐ATG into ATC (ΔATG) resulted in the loss of Tr‐NfC/EBPα expression, while converting uORF‐ATG into an optimal Kozak sequence (GCC ATG C ‐> GCC ATG G) (ATG^Opt^) caused upregulation of Tr‐NfC/EBPα, confirming that its expression is dependent on uORF translation. Mutation of the Ext‐CTG codon into CTC (ΔCTG) resulted in the loss of Ext‐NfC/EBPα, proving its use as an alternative initiation codon. Finally, mutation of the double ATG (ΔATG^Tr^), believed to be the initiation sites for the Tr‐NfC/EBPα isoform, caused a shift in expression to a smaller protein, likely due to ribosomes scanning to the next available AUG codon in the *NfCEBPA* reading frame. This confirmed that the double ATGs serve as initiation sites for Tr‐NfC/EBPα expression. The expression of single Tr‐NfC/EBPα proteins is shown for reference (Figure 3b and Figure S1).

In summary, the regulation of NfC/EBPα protein isoform expression relies on the same structural elements in the NfCEBPA mRNA and follows the same regulatory rules involving a *cis*‐regulatory uORF, as demonstrated in human, rat, mouse, and chicken orthologs (Calkhoven et al. 1994, 2000; Muller et al. 2010).

### 2.3 Removal of the ΔuORF Enhances NfC/EBPα Transactivation Activity

**FIGURE 4:**
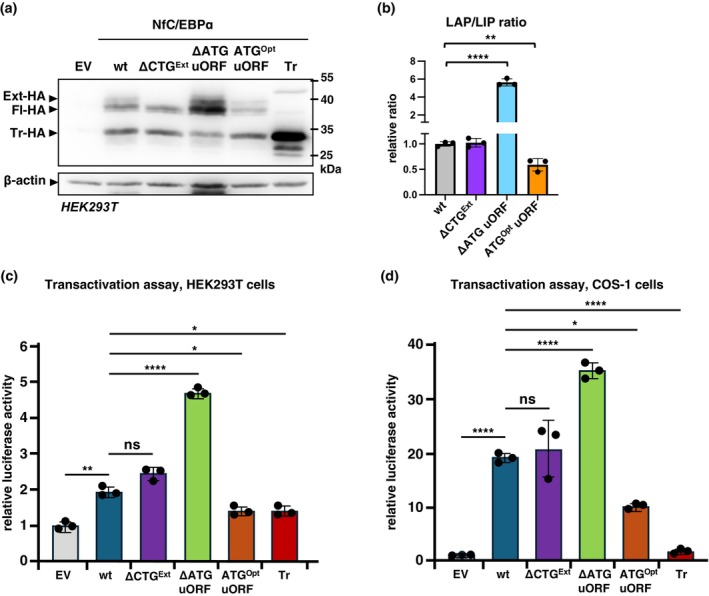
Impact of the CEBPA‐ΔuORF mutation on NfC/EBPα transactivation activity. (a) Immunoblot analysis of NfC/EBPα construct expression in HEK293T cells, with β‐Actin serving as a loading control. (b) Bar graph showing quantifications of the relative changes in LAP/LIP‐isoform ratios by chemiluminescence digital imaging from three biological replicates (panel a and Figure S1b). Values are mean ± SD; significance was determined by Student's *t*‐tests, **p < 0.01, ****p < 0.0001. (c, d) Bar graphs showing the fold induction of the 2xC/EBP‐binding sites luciferase reporter in HEK293T cells (c) or COS‐1 cells (d and Figure 3b) with coexpression of various NfC/EBPα constructs: Wild‐type (wt), ΔCTG (CTG for Ext‐NfC/EBPα removed), ΔATG‐uORF (removal of the uORF), ATG^Opt^‐uORF (optimizing uORF function), and Tr‐NfC/EBPα only. Statistical differences were determined by one‐way ANOVA with multiple comparisons. Error bars represent ± SD. *p < 0.05, **p < 0.01, ****p < 0.0001.

To investigate whether the truncated NfC/EBPα isoform, expressed from the NfCEBPA mRNA, affects the overall transactivation potential of NfC/EBPα proteins, we co‐transfected HEK293T or COS‐1 cells with a luciferase reporter vector containing two consensus C/EBP binding sites and the following expression vectors: wild‐type NfC/EBPα (wt); a mutant with the upstream CTG initiation codon removed (ΔCTG), preventing expression of Ext‐NfC/EBPα; a mutant with the uORF removed (ΔATG^uORF^), reducing Tr‐NfC/EBPα in favor of Fl‐NfC/EBPα; a mutant with an optimized Kozak sequence at the uORF initiation site (ATG^uORFopt^), increasing Tr‐NfC/EBPα at the cost of Fl‐NfC/EBPα; and an expression vector for Tr‐NfC/EBPα only (Figure 3b,c and Figure S1a, and Figure 4a,b and Figure S1b). The ΔCTG mutation did not alter the transactivation potential compared to the wild‐type NfC/EBPα in both cell lines (Figure 4c,d), likely due to the distinct function of Ext‐NfC/EBPα in Pol I‐mediated rDNA transcription in the nucleolus (Muller et al. 2010). The ΔATG^uORF^ mutation resulted in a significant increase in NfC/EBPα transactivation potential compared to wild‐type NfC/EBPα, likely due to diminished expression of the truncated isoform, which normally competes with the extended‐ and full‐length isoforms for DNA binding. In contrast, the ATG^uORFopt^ mutant showed decreased transactivation potential, reflecting the increased expression of the inhibitory truncated isoform. Finally, expression of the Tr‐NfC/EBPα alone was unable to activate the transcription reporter above the basic empty vector level (EV) (Figure 4c,d). Thus, as observed in other vertebrates, the loss of uORF‐mediated Tr‐NfC/EBPα isoform expression leads to enhanced NfC/EBPα activity.

### 2.4 Enhanced NfC/EBPα Activity Extends Lifespan in Male *N. furzeri*

**FIGURE 5:**
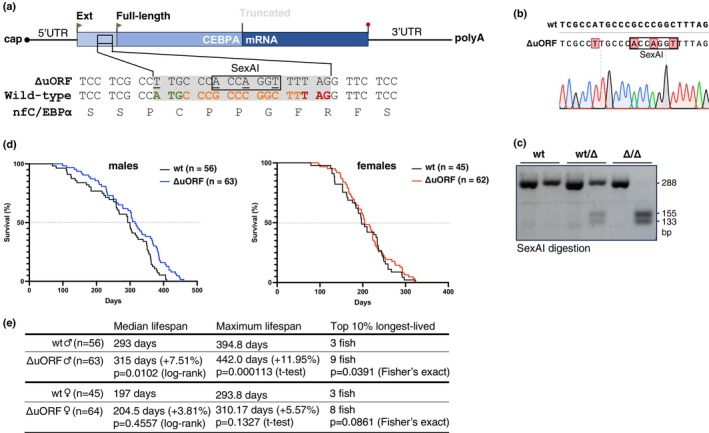
CEBPA‐ΔuORF mutation extends the lifespan of male killifish. (a) Schematic representation of NfCEBPA mRNA with corresponding cDNA sequences showing mutations that ablate the uORF and introduce a SexAI restriction site for genotyping purposes. Wild‐type NfC/EBPα sequences show the +2 out‐of‐frame uORF sequence highlighted in color. The NfC/EBPα amino acid sequence shown below reveals that the Ext‐NfC/EBPα sequence is not affected by the mutation. (b) Sequencing result of genomic DNA from ΔuORF homozygous mutant fish. (c) PCR analysis of *NfCEBPA* genomic DNA from the wild‐type, heterozygous, and homozygous ΔuORF mutants. (d) Survival curves of lifespan experiments for wild‐type (wt) or *NfCEBPA* ^*ΔuORF*^ fish, separated by sex. (e) Table summarizing the median lifespan, maximum lifespan, and top 10% longest‐lived fish, with statistical analysis as indicated.

In the *N. furzeri* strain ZMZ1001, we used CRISPR/Cas9 genome editing to disrupt uORF functionality by mutating the uORF‐ATG to TTG, creating the *NfCEBPA* ^*ΔuORF*^ strain (Figure 5a). Successful mutation was confirmed by sequencing (Figure 5b) and PCR analysis (Figure 5c). Heterozygous males and females were bred to generate homozygous offspring.

To examine lifespan differences between wild‐type fish and *NfCEBPA* ^*ΔuORF*^ mutants, we monitored offspring from the same heterozygous mating pairs across six female and seven male cohorts. In total, 45 female wt, 62 female ΔuORF, 56 male wt, and 63 male ΔuORF fish were observed from an age of 8 to 10 weeks until natural death or termination based on humane endpoint criteria. Analysis of the combined male cohorts revealed a significantly extended lifespan for *NfCEBPA* ^*ΔuORF*^ males compared to wild‐type males (Figure 5d,e). Specifically, the median lifespan increased by 7.5%, whereas the maximum lifespan, defined as the mean lifespan of the top 10% longest‐lived fish in each cohort, was extended by nearly 12%. Among the top 10% of longest‐lived male fish, nine carried the ΔuORF mutation, compared to only three wild‐type fish. No significant difference in lifespan was observed between the female wild‐type and *NfCEBPA* ^*ΔuORF*^ fish (Figure 5d,e). Finally, comparing wild‐type killifish, females had shorter lifespans than males, with median lifespans of 197 and 293 days for females and males, respectively (Figure 5e). These findings suggest that the ΔuORF mutation, which increases NfC/EBPα transactivation potential, confers a lifespan advantage to male fish of the *N. furzeri* ZMZ1001 strain, particularly during later stages of life, whereas females do not show a similar benefit.

### 2.5 Enhanced NfC/EBPα Activity Partially Improves Healthspan in *N. furzeri*

**FIGURE 6:**
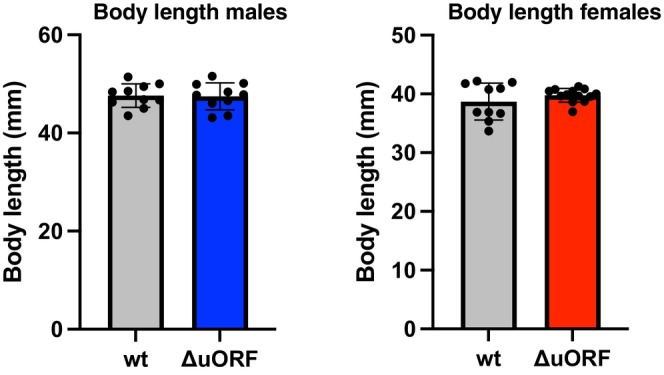
The *NfCEBPA* ^*ΔuORF*^ mutation does not affect body size. At the left, data from males that died between 293 and 321 days of age, *n* = 10 for both wild‐type (wt) and *NfCEBPA* ^*ΔuORF*^ (ΔuORF) fish. At the right, data from females that died between 176 and 204 days of age, *n* = 10 for wild‐type (wt) and *n* = 13 for *NfCEBPA* ^*ΔuORF*^ (ΔuORF) fish. Values are mean ± SD. Statistical analysis using Student's *t*‐tests showed no significant differences.

Compared to wild‐type fish, the *NfCEBPA* ^*ΔuORF*^ fish exhibited no visible development abnormalities and showed no differences in body length among males that died between 293 and 321 days of age and females that died between 176 and 204 days (Figure 6). To assess the healthspan in the lifespan cohorts, we conducted twice‐daily visual inspections of each fish, documenting changes in appearance and behavior. Tables 1 and 2 provide an overview of the outcomes, including spontaneous mortality, humane endpoint‐based terminations, aging‐related phenotypes, and swimming abnormalities, some of which are discussed in further detail below.

**TABLE 1:** Mortality, humane endpoint termination, and aging phenotypes.

Sex	Genotype	No.	Found dead % (no.)	Terminated % (no.)	Weight loss % (no.)^a^	Dropsy % (no.)^a^	Rectal prolapse % (no.)^a^	Color loss % (no.)^b^	Tumors % (no.)^b^
Male	Wild‐type	56	28.6 (16)	71.4 (40)	12.5 (7)	25.0 (14)	—	37.5 (21)	10.7 (6)
	ΔuORF	63	28.6 (18)	71.4 (45)	20.6 (13)	28.6 (18)	—	33.3 (21)	4.8 (3)
Female	Wild‐type	45	64.4 (29)	35.6 (16)	2.2 (1)	62.5 (11)	8.9 (4)	13.3 (6)	17.8 (8)
	ΔuORF	62	59.7 (37)	40.3 (25)	4.8 (3)	64.0 (16)	6.5 (4)	17.7 (11)	8.1 (5)

^a^ Observed near or at time of humane endpoint termination.

^b^ Observed throughout life.

**TABLE 2:** Abnormal swimming phenotypes.

Sex	Genotype	No.	Belly sliding % (no.)^a^	Floating % (no.)	Upside down % (no.)	Unbalanced % (no.)	Sideways % (no.)	Total % (no.)
Male	Wild‐type	56	21.4 (12)	5.4 (3)	10.7 (6)	3.7 (2)	16.1 (9)	57.2 (32)
	ΔuORF	63	20.6 (13)	4.8 (3)	3.2 (2)	1.6 (1)	11.1 (7)	41.3 (26)
Female	Wild‐type	45	—	6.7 (3)	2.2 (1)	6.7 (3)	8.9 (4)	24.4 (11)
	ΔuORF	62	3.2 (2)	—	—	—	12.9 (8)	16.1 (10)

^a^ Persistent belly sliding for a duration of 7 days or more.

**FIGURE 7:**
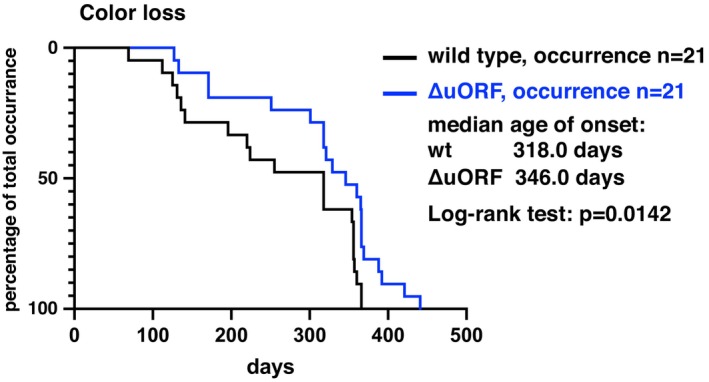
Delayed color loss in *NfCEBPA* ^*ΔuORF*^ male fish. The curves represent a progressive increase in the percentage of male fish exhibiting color loss throughout their lifespan.

Among the spontaneously deceased killifish, approximately one‐third were males and two‐thirds were females. In contrast, two‐thirds of the males were euthanized based on humane endpoint criteria, compared to only one‐third of the females (Table 1). The humane endpoint indicators included severe deterioration in general appearance, indicating imminent death, such as abnormal swimming positions and poor body condition, severe abdominal distension (dropsy) with raised scales, visible tumors with progressive growth, often accompanied by poor body condition, markedly reduced movement, and lack of food intake. At the end of life, males exhibited severe weight loss more frequently than females: 12.50% in wild‐type and 20.64% in *NfCEBPA* ^*ΔuORF*^ males compared to 2.22% in wild‐type females and 4.84% in *NfCEBPA* ^*ΔuORF*^ females. These data suggest that the *NfCEBPA* ^*ΔuORF*^ mutation promotes weight loss in aging fish. The dropsy phenotype was more common in females than in males, with no significant differences between the genotypes. Rectal collapse was observed exclusively in females and occurred slightly more frequently in wild‐type fish than in *NfCEBPA* ^*ΔuORF*^ fish. The incidence of visible tumors was significantly reduced in *NfCEBPA* ^*ΔuORF*^ fish, with 10.71% of wild‐type males and 17.78% of wild‐type females affected compared to 4.76% and 8.07% in *NfCEBPA* ^*ΔuORF*^ males and females, respectively. Male killifish, which are naturally more colorful than females, showed age‐related loss of color. Although a similar percentage of males showed color loss, this was delayed in the *NfCEBPA* ^*ΔuORF*^ males (Figure 7).

**FIGURE 8:**
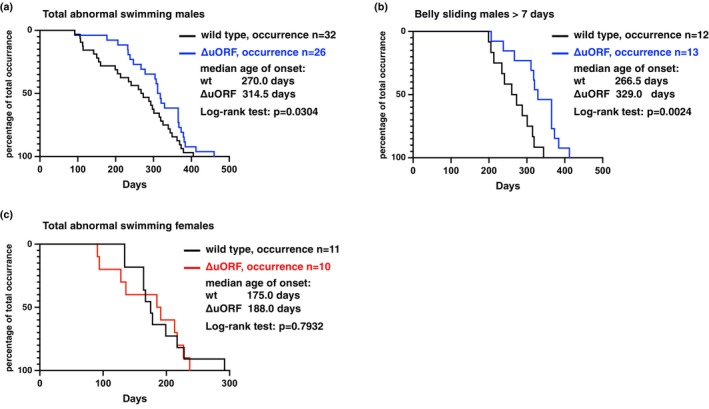
CEBPA‐ΔuORF mutation delays abnormal swimming behavior in males. The curves represent the progressive increase in the percentage of fish exhibiting (a) total abnormal swim behaviors in males, (b) belly‐sliding in males, and (c) total abnormal swimming in females.

Fish can develop swim bladder dysfunctions due to infections, environmental causes, and aging, which manifest as abnormal swimming behaviors. Table 2 categorizes five distinct phenotypes: belly sliding, where fish struggle to lift from the bottom of the tank, a condition that, although sometimes observed in young fish and typically resolves within 3 days, becomes more persistent (over 7 days) in older fish; floating, where fish remain at the water surface, often in abnormal body positions; upside down, with the belly facing upwards; unbalanced, meaning that the position of the head is either lower or higher than the tail; and sideways, when fish swim tilted to one side at various angles. Overall, the frequency of abnormal swimming behaviors was significantly higher in males than in females (Table 2). Additionally, the incidence of these behaviors was lower in *NfCEBPA* ^*ΔuORF*^ males and slightly lower in *NfCEBPA* ^*ΔuORF*^ females, compared to wild‐type fish (Table 2). In *NfCEBPA* ^*ΔuORF*^ males, the occurrence of abnormal swimming behaviors was significantly delayed compared to wild‐type males (Figure 8a), which was also observed for belly sliding alone as the largest contributor to abnormal swimming (Figure 8b). No significant differences in timing were found among the female fish (Figure 8c). Together, these results suggest that while swim bladder function is more susceptible to age‐related decline in male fish, this decline is delayed in *NfCEBPA* ^*ΔuORF*^ males, indicating a potential protective effect of the ΔuORF mutation on swim bladder function as fish age.

### 2.6 Differential Gene Regulation in *NfCEBPA* ^*ΔuORF*^ Fish

Given that the ΔuORF mutation affects the function of a transcription factor, changes in the downstream gene regulation are anticipated. We performed comparative genome‐wide transcriptome analysis of liver, skin, brain, and muscle tissues from *NfCEBPA* ^*ΔuORF*^ and wild‐type male fish from the lifespan cohorts at humane endpoint termination (*NfCEBPA* ^*ΔuORF*^, aged 365, 367, and 408 days; wild‐type, aged 363, 367, and 373 days). In the liver of *NfCEBPA* ^*ΔuORF*^ *fish*, 505 genes were upregulated and 205 downregulated in *NfCEBPA* ^*ΔuORF*^ compared to wild‐type fish. In the skin, 125 genes were upregulated and 80 downregulated; in muscle, 219 were upregulated and 152 downregulated; and in the brain, 49 genes were upregulated and 9 downregulated (Table S1).

**FIGURE 9:**
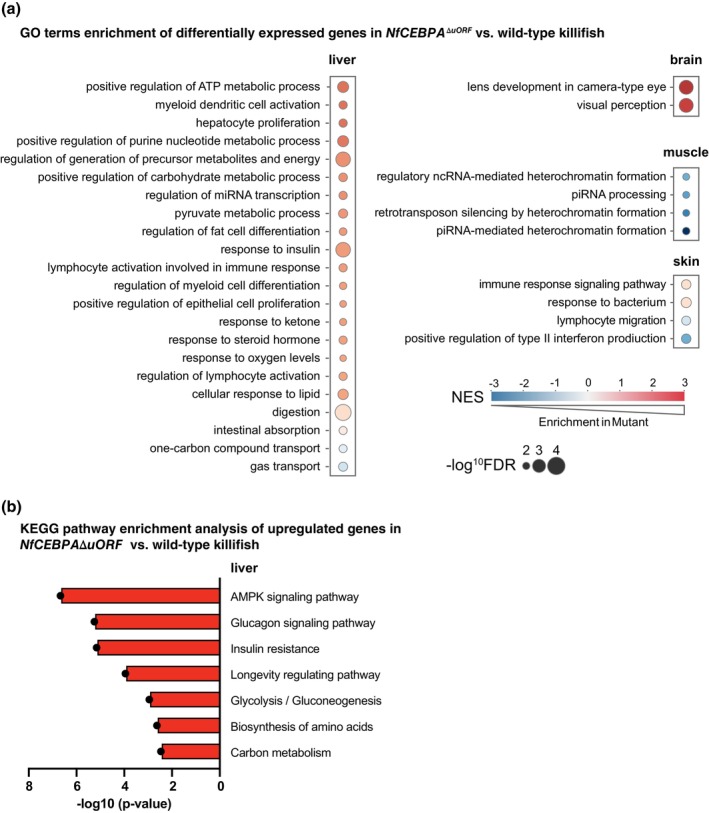
GO‐term and KEGG pathway analysis of differentially regulated genes in *NfCEBPA* ^ΔuORF^ versus wild‐type killifish. (a) Normalized Enrichment Scores (NES) are represented by color intensity: Red indicates upregulation and blue indicates downregulation. False discovery rates (FDR) are shown as −log10 with values 2, 3 and 4 indicating increasing levels of significance. (b) KEGG pathway enrichment analysis of upregulated genes in the liver. Bars represent *p*‐values (−log10) as shown on the *x*‐axis. Only pathways with enrichment scores > 2.5 are shown.

Gene ontology (GO) term enrichment and functional annotation clustering using Kyoto Encyclopedia of Genes and Genomes (KEGG) pathway analysis of the differentially expressed genes showed that a subset of the upregulation genes in liver is enriched in biological processes associated with improved healthspan and lifespan (Figure 9a,b). For the GO term analysis these include metabolic processes, cell proliferation and differentiation, and immune response regulation (Figure 9a and Table S2). The most significant enrichment (−log_10_FDR ≥ 4) was observed for the GO terms “positive regulation of ATP metabolic processes,” “regulation of generation of precursor metabolites and energy,” “response to insulin,” and “digestion.” In contrast, differentially expressed genes in the brain, muscle and skin were associated with fewer significantly enriched GO terms. In the skin and muscle, only downregulated genes contributed to GO term enrichment, whereas in the brain, only upregulated genes were linked to the enriched GO terms. Functional KEGG pathway annotation clustering of the upregulated genes in the liver revealed in pathways such as “longevity regulating pathway” and “AMPK signaling pathway” (Figure 9b and Table S1). These data suggest that, at least in the liver, metabolic and proliferative tissue functions known to decline with aging are better maintained in male *NfCEBPA* ^*ΔuORF*^ fish compared to wild‐type fish, and that pro‐longevity pathways are induced.

## 3 Discussion

Here, we demonstrate that translation of the *NfCEBPA* gene transcript in the African turquoise killifish *Nothobranchius furzeri* produces three distinct protein isoforms: extended (Ext)‐, full‐length (Fl)‐, and truncated (Tr)‐NfC/EBPα. By identifying the translation initiation codons involved, we established that Tr‐NfC/EBPα expression depends on the integrity of a small upstream open reading frame (uORF). These features mirror those reported for *CEBPA* orthologs in mice, rats, chickens, and humans, underscoring the evolutionary conservation of the *CEBPA* genes and their C/EBPα protein products across a wide range of vertebrates. Disruption of the *NfCEBPA* uORF in killifish by CRISPR/Cas9 mediated genomic modification significantly extended the median lifespan of the male *NfCEBPA* ^*ΔuORF*^ fish by +7.51% and the maximum lifespan by +11.95%, along with delays and reductions in various age‐related phenotypes. Notably, female *NfCEBPA* ^*ΔuORF*^ killifish did not show similar improvements. Despite testing multiple anti‐C/EBPα antibodies targeting epitopes that partially overlap with the NfC/EBPα sequences, none demonstrated sufficient reactivity against endogenous NfC/EBPα. However, numerous experiments with C/EBPα and C/EBPβ of other species have consistently demonstrated that uORF‐dependent regulation operates identically in both cell culture and whole organisms.

We observed a generally longer lifespan in males than in females, which contrasts with previous studies reporting no sex‐based difference in lifespan (Graf et al. 2010; Valenzano et al. 2006). Notably, our female cohort exhibited a high incidence of the dropsy phenotype and an increased frequency of spontaneous death. These findings suggest that disease susceptibility or potentially unfavorable group‐housing conditions for females may have contributed to their reduced lifespan. It is possible that these factors masked or outweighed lifespan effects associated with the *NfCEBPA* ^*ΔuORF*^ mutation. However, the sex‐specific effects on health and lifespan observed in *NfCEBPA* ^*ΔuORF*^ killifish remind us of the findings in mice carrying a mutation in the uORF of the related *CEBPB* gene (*CEBPB* ^*ΔuORF*^ mice). From CEBPB mRNA, three protein isoforms are translated into C/EBPβ‐LAP1/2 and the N‐terminally truncated isoform C/EBPβ‐LIP (Calkhoven et al. 2000; Wethmar, Begay, et al. 2010). Disruption of the *CEBPB* uORF in mice enhances C/EBPβ transactivating activity, resulting in improved metabolic profiles similar to those observed under calorie restriction. In addition, the mutation helps preserve motor coordination and immune functions in older *CEBPB* ^*ΔuORF*^ mice compared to their wild‐type littermates in both sexes. However, an extension of the median lifespan of 20.6% and a maximum lifespan of 10% was only observed in females (Muller et al. 2018, 2022; Zidek et al. 2015). Additionally, impaired demethylation of C/EBPβ‐regulated super‐enhancers, which prevents C/EBPβ binding, has been linked to premature aging in mice (Schafer et al. 2018). Notably, aging‐related upregulation of Tr‐C/EBPα (p30) levels and DNA‐binding activity has been observed in aged livers of mice (Hsieh et al. 1998). Similarly, increased expression of C/EBPβ‐LIP during aging has been reported in human adipose tissues (Karagiannides et al. 2001) and in mouse liver (Muller et al. 2018; Timchenko et al. 2006). These findings suggest a potential role for the truncated C/EBP isoforms in age‐related conditions; however, direct experimental evidence supporting this role is still lacking. Together with the data presented here, these findings underscore the essential role of both C/EBPα and C/EBPβ in maintaining health and resilience throughout aging (Niehrs and Calkhoven 2020).

Within the C/EBP transcription factor family, only *CEBPA* and *CEBPB* share the unique features of being intronless, containing a cis‐regulatory uORF, and utilizing three alternative translation initiation sites to produce three distinct protein isoforms of C/EBPα and C/EBPβ (Ramji and Foka 2002; Wethmar, Smink, and Leutz 2010). Comparative analysis of mRNA sequences across vertebrates has revealed remarkable conservation in the distribution, position, and relative strength of these initiation sites, highlighting their evolutionary importance. Studies have shown that uORF‐driven expression of truncated‐C/EBPα (p30) and C/EBPβ‐LIP are tightly regulated by the mTORC1‐4E‐BP and eIF2α kinase pathways (Calkhoven et al. 2000; Zidek et al. 2015), linking these C/EBPs to cellular nutrient and stress signaling. Either inhibition of mTORC1 signaling or activation of eIF2α kinases results in the suppression of truncated C/EBPα (p30) and C/EBPβ‐LIP expression. These shared regulatory mechanisms suggest that both C/EBPα and C/EBPβ act as integrators of nutrient, growth factor, and stress signals to orchestrate a transcriptional response (Niehrs and Calkhoven 2020). Furthermore, Fl‐C/EBPα function is connected to Acetyl‐CoA and NAD^+^ metabolism through its acetylation by the acetyltransferase p300 and deacetylation by SIRT1 (Zaini et al. 2018). In response to glucose deprivation, increased NAD+ levels activate SIRT1, which deacetylates C/EBPα. Hypoacetylated C/EBPα shifts the downstream transcriptional response towards genes involved in mitochondrial biogenesis and respiration.

Notably, the most prominent phenotype in the *NfCEBPA* ^*ΔuORF*^ fish observed here was a reduced incidence of visible cancers, suggesting a similar prominent role of C/EBPα in cancer development, as has been observed in *CEBPB* ^*ΔuORF*^ mice. C/EBPβ‐LIP deficiency reduces tumor incidence (Muller et al. 2018), whereas its overexpression increases tumor incidence in mice (Begay et al. 2015). Furthermore, C/EBPβ‐LIP is highly expressed in several human cancers, including breast cancer, ovarian cancer, colorectal cancer, and anaplastic large cell lymphoma (Jundt et al. 2005; Quintanilla‐Martinez et al. 2006; Rask et al. 2000; Sundfeldt et al. 1999; Zahnow et al. 1997), and C/EBPβ‐LIP overexpression induces cellular transformation in vitro and in vivo (Calkhoven et al. 2000; Zahnow et al. 1997). Mechanistically, C/EBPβ‐LIP supports oncogenesis through various pathways. For instance, it induces a shift towards cancer‐specific metabolic reprogramming by enhancing glycolysis and mitochondrial respiration, mediated in part by the regulation of the let‐7/LIN28B circuit (Ackermann et al. 2019). Additionally, C/EBPβ‐LIP promotes the activity of the malate–aspartate shuttle, ensuring NADH/NAD^+^ homeostasis in cancer cells (Ackermann et al. 2023). Beyond metabolic reprogramming, C/EBPβ‐LIP has been shown to stimulate cancer cell migration and invasion in cell culture, suggesting a crucial role in metastasis (Matherne et al. 2023; Sterken et al. 2022).

The truncated isoform of C/EBPα (c) has also been implicated in cancer development. In acute myeloid leukemia (AML), mutations in the *CEBPA* gene frequently generate premature stop codons, preventing the expression of the extended (Ext‐C/EBPα) and full‐length (FL‐C/EBPα/ C/EBPα‐p42) isoforms, while leaving the C/EBPα‐p30 isoform unaffected (Nerlov 2004). Experimental studies in mice demonstrated that replacing the *CEBPA* allele with sequences exclusively expressing C/EBPα‐p30 induces AML with full penetrance, suggesting that C/EBPα‐p30 acts as an oncogene (Bereshchenko et al. 2009; Kirstetter et al. 2008). Furthermore, overexpression of C/EBPα‐p30 in 3T3‐L1 adipoblasts induces cellular transformation (Calkhoven et al. 2000). Mechanistically, C/EBPα‐p42 exerts strong anti‐proliferative activity, which is dominant over the loss of tumor suppressors, such as p53 or Rb (Hendricks‐Taylor and Darlington 1995; Muller et al. 1999). In contrast, the truncated isoform lacks this tumor‐suppressive function, and by competing with C/EBPα‐p42 for binding to target gene promoters and enhancers, it facilitates cell proliferation. The specific downstream mechanisms and physiological processes by which C/EBPα induces cell proliferation and contributes to cancer development require further investigation.

Both male and female *NfCEBPA* ^*ΔuORF*^ fish showed a notable reduction in abnormal swimming behavior compared to their wild‐type counterparts. These phenotypes, which are often linked to swim bladder dysfunction, can arise from infections, environmental influences, or age‐related decline. The reduced incidence of such behaviors in mutant fish suggests an enhanced resilience to these challenges. Although this finding is limited in scope, it aligns with observations in *CEBPB* ^*ΔuORF*^ male mice, which display increased resistance to ulcerative dermatitis, a condition prevalent in C57BL/6 mice (Muller et al. 2018). Additionally, older *CEBPB* ^*ΔuORF*^ mice display remarkably preserved motor coordination and a more youthful immune profile, indicated by increased memory/naïve T‐cell ratios, highlighting the broader health benefits conferred by uORF disruption in these transcription factors.

In this study using killifish and in studies using mice, sex‐specific phenotypes in response to genetic modifications were observed. Such differences in responses to mutations or treatments are often poorly understood. In laboratory animal models, it is important to consider that genetic background plays a significant role. For example, the genetic makeup of the *N. furzeri* ZMZ1001 strain used in this study may substantially contribute to the observed sex‐specific responses. Similarly, studies in genetically diverse mice under various calorie restriction regimens have revealed a strong dependence on genetic background for phenotypic variation. In addition, hormonal interactions, both systemic and at the level of nuclear hormone receptor–C/EBP interactions, likely play essential roles in mediating sex‐specific effects.

Comparative transcriptome analysis revealed 710 differentially regulated genes in the liver of *NfCEBPA* ^*ΔuORF*^ fish, the majority of which were upregulated. This likely reflects the enhanced transactivation capacity due to the loss of the inhibitory Tr‐NfC/EBPα. Consistent with this, our previous study on aged *CEBPB* ^*ΔuORF*^ mice, which exhibit enhanced C/EBPβ transactivation capacity, also revealed a predominance of upregulated genes in the liver (Muller et al. 2018). GO term analysis of the *NfCEBPA* ^*ΔuORF*^ liver transcriptome revealed upregulation of pathways potentially relevant to healthspan and lifespan regulation, including those involved in metabolic processes and cellular proliferation and differentiation. Maintenance of metabolic performance is widely regarded as a key determinant of healthy aging (Lopez‐Otin et al. 2016). Moreover, genes associated with proliferation and the cell cycle are typically downregulated with age in the liver, brain, and skin of *N. furzeri* (Baumgart et al. 2016). In contrast, increased expression of proliferation‐related genes has been linked to a younger age in *N. furzeri* muscle (Xu et al. 2023), possibly reflecting an enhanced regenerative capacity of the tissue. Furthermore, functional annotation clustering using KEGG pathway analysis of the upregulated liver genes revealed enrichment of the longevity regulating and AMP‐activated protein kinase (AMPK) signaling pathways in the *NfCEBPA* ^*ΔuORF*^ fish. AMPK is a key intracellular energy sensor, activated by low‐energy levels through an increased AMP:ATP ratio. Its pro‐longevity function is well established across species, from lower vertebrates to mammals, and has recently been shown in *N. furzeri* (Astre et al. 2023; Burkewitz et al. 2014).

In the skin of *NfCEBPA* ^*ΔuORF*^ fish, we observed reduced expression of genes associated with the GO terms “immune response‐activating signaling pathways” and “positive regulation of type II interferon production” compared to wild‐type. This suggests that the age‐related increase in low‐grade inflammation, known as “inflammaging,” is attenuated in the mutant fish.

Furthermore, the expression of FoxO transcription factor genes, *foxo1* and *foxo3*, which are known to promote health and longevity across species (Martins et al. 2016) was upregulated in the livers of the *NfCEBPA* ^*ΔuORF*^ fish. In addition, several genes involved in the cellular stress response showed increased expression. These include heat shock factor genes (*hspa1b, hspa1l, hspb6, and hsp90aa1*), DNA damage response genes (*ddit4, sesn1, gadd45a, gadd45b, and gadd45g*), the unfolded stress response transcription factor gene *xbp1*, and the oxidative stress response gene *hif1a*. Upregulation of stress response genes has previously been observed in various longevity models (Soo et al. 2023) suggesting that enhanced resilience may contribute to improved healthspan and lifespan by protecting against age‐associated cellular decline.

Parallels can be drawn between *NfCEBPA* ^*ΔuORF*^ fish and the *CEBPB* ^*ΔuORF*^ mouse model. In both, the GO term “lymphocyte activation” is enriched in the liver transcriptome. While the (patho)physiological significance of this finding needs further clarification, it raises the possibility of a more efficient lymphocyte‐mediated clearance of senescent cells in the liver. Additionally, both models show increased expression of genes involved in lipolysis and β‐oxidation in the liver, such as *lpl, cpt1a*, and *ppara* in killifish, and similar genes in mice (Zidek et al. 2015). This suggests enhanced fatty acid oxidation, a metabolic shift that is also observed in calorie restriction models (Bruss et al. 2010), which probably contributes to the improved healthspan and lifespan observed in both species.

However, some results are more challenging to interpret. For example, in the muscle of *NfCEBPA* ^*ΔuORF*^ fish, we observed the downregulation of genes associated with GO terms related to heterochromatin formation and retrotransposon silencing by heterochromatin formation. Aging is accompanied by a partial loss of heterochromatin‐mediated silencing, leading to the activation of retrotransposons (Gorbunova et al. 2021). Understanding the potential implications of this downregulation in muscle function requires further investigation. Similarly, insulin resistance emerged as an enriched KEGG pathway based on functional annotation analysis of upregulated liver genes in *NfCEBPA* ^*ΔuORF*^ fish. This finding contrasts with the increased insulin sensitivity typically observed in several established longevity models (Bartke and Brown‐Borg 2004; Dos Santos et al. 2024). Notably, however, treatment with rapamycin extends both healthspan and lifespan in mice despite inducing insulin resistance (Lamming et al. 2012). At this stage, it remains unclear whether the gene expression profile observed in the mutant fish corresponds to an actual decrease in hepatic insulin sensitivity.

The transcriptomic samples derived from the lifespan cohort vary both in age and health status, which may confound the interpretation of differential gene expression attributed to the *NfCEBPA* ^*ΔuORF*^ mutation. To more accurately dissect the direct gene regulatory effects of the *NfCEBPA* ^*ΔuORF*^ mutation, more controlled or reductionistic experimental systems are required. For instance, fibroblast cell lines derived from killifish or the use of CRISPR/Cas9‐mediated genome editing to generate *CEBPA‐*mutant cell lines could offer more controlled experimental settings to study the effects of increased C/EBPα transcription factor function on different target genes.

This study aimed to confirm the evolutionary conservation of C/EBPα regulation, focusing on its primary mRNA structure, while also providing novel insights into how altering NfC/EBPα protein isoform expression impacts health and lifespan. These findings, along with research on C/EBPβ in mice, underscore the importance of further investigation into the role of C/EBP transcription factors in promoting resilience against age‐related conditions and diseases.

The parallels between C/EBPα and C/EBPβ suggest shared and distinct roles in the regulation of cellular processes that contribute to aging and longevity. Key unanswered questions remain, including identifying the specific biological processes uniquely regulated by C/EBPα or C/EBPβ, and those where their functions overlap. Investigating how these transcription factors integrate nutrient and stress signaling pathways, such as those involving mTORC1, eIF2α‐kinases, and sirtuins, could reveal how environmental and metabolic cues influence their activity. Additionally, the interplay between C/EBPs and other transcriptional regulators, such as nuclear hormone receptors, deserves more attention to understand their roles in cellular homeostasis.

Although it is challenging to pharmacologically target transcription factors because of their structural properties, the signaling pathways that regulate their activity and/or expression represent promising therapeutic avenues (Zaini et al. 2017). Identifying upstream modulators of C/EBP function and dissecting their mechanistic roles is essential for developing strategies to enhance resilience against aging and age‐related diseases. Finally, expanding research in diverse model organisms and human systems is also critical for translating these findings into clinical applications.

## 4 Materials and Methods

### 4.1 DNA Constructs

The *CEBPA* cDNA from *N. furzeri* ZMZ1001 strain, corresponding to the 5′ UTR and coding region (wt‐C/EBPα), and the ∆5′ UTR construct lacking the 5′ UTR, were amplified using genomic DNA and fused at their C‐terminus to the HA epitope. They were cloned into pCDNA3.1 (Invitrogen) via Gibson Assembly (NEBuilder HiFi DNA Assembly kit, NEB) using primers listed below. Fl‐, Tr‐, ∆ATG‐uORF‐, ATGOpt‐uORF‐, and ∆ATGTr‐C/EBPα pCDNA3.1 constructs were generated by whole plasmid PCR amplification, phosphorylation, and blunt ligation. The ∆CTGExt‐C/EBPα construct was generated by exchanging the N‐terminus of wild‐type C/EBPα using PCR and restriction enzymes NheI/AgeI. To subclone expression constructs into pSG5, wild‐type C/EBPα and mutants were PCR‐amplified from pCDNA3.1‐based constructs and cloned into pSG5 (Stratagene) via EcoRI. All constructs were sequence‐verified by Sanger sequencing.

**Table:** 

Construct	Forward primer	Reversed primer
wt‐C/EBPα backbone	TACCCATACGATGTTCCAGAT TACGCTTGATCAGCCTCGACT GTGCCTTCTAGTTG	GCTGCGGACAGGAGCCGACCG GAACCAGAGGGTGGCTAGCCA GCTTGGGTC
wt‐C/EBPα insert	CTCTGGTTCCGGTCGGCTCC	TCAAGCGTAATCTGGAACATCG TATGGGTAGCTGCCCATGGGTT TGAACGAC
Δ5′UTR‐C/EBPα backbone	TACCCATACGATGTTCCAGAT TACGCTTGATCAGCCTCGACT GTGCCTTCTAGTTG	ACAGGTTCGAGAGCTCCATGG TGGCTAGCCAGCTTGG
Δ5′UTR‐C/EBPα insert	AGCTGGCTAGCCACCATGGAG CTCTCGAACCTGTACGAGG	TCAAGCGTAATCTGGAACATCG TATGGGTAGCTGCCCATGGGTT TGAACGAC
Fl‐C/EBPα	GCCATGGAGCTCTCGAACCTG TACG	GAACCTAAAGCCGGGCGGG
Tr‐C/EBPα	ATGATGAATGGAGAGTATGAC TGCGGTCC	GGTGGCTAGCCAGCTTGGGTC
ΔATG‐uORF‐C/EBPα	ATCCCCGCCCGGCTTTAGGTT CTCC	GGCGAGGAGCTCCGGAAATCCA
ATGOpt‐uORF—	ATGGCCGCCCGGCTTTAGGTT C	GGCGAGGAGCTCCGGAAATCCA
ΔATG^Tr^	CCACAGCAGCTCTACATCCCT AACTACATCGAGTCCAAGCTG GAGCCGTTCTAC	ACCCGGACCGCAGTCGTACTCT CCGTTGATGATCTTGACTTTGTC CTGTCGCGAGTTGT
ΔCTG^Ext^ mutation	GACTTTCCAAACTCGATTTCC GGAGCTC	GAGCTCCGGAAATCGAGTTTGG AAAGTC
ΔCTG^Ext^ outer primers	GACGCAAATGGGCGGTAGGC GTG	CCGGGTTCAGGTCCTCGTCATC C
for subcloning into pSG5	ATGATGAATTCGGAGACCCAA GCTGGCTAGC	CTGCCGAATTCTCAAGCGTAAT CTGGAACATCG

### 4.2 Cells

COS‐1 cells were cultured in DMEM/F12 with 5% FCS, 10 mM HEPES, and antibiotics. HEK293T and Hepa1‐6 cells were cultured in DMEM with 10% FCS, 10 mM HEPES, and antibiotics. All cells were maintained at 37°C with 5% CO_2_.

### 4.3 Transfection

COS‐1 cells were transfected with 5 μg pSG5‐based constructs using the DEAE‐dextran/chloroquine method. HEK293T cells were transfected with 2.5 μg pCDNA3.1 constructs via calcium phosphate precipitation. Hepa1‐6 cells were transfected with 4 μg pCDNA3.1 constructs using 12 μL Fugene HD (Promega), following the manufacturer's protocol.

### 4.4 Luciferase Reporter Assay

Cells were seeded at 5000 (HEK293T) or 6000 (COS‐1) cells/well in a 96‐well plate. After 24 h, they were transfected with 600 ng pCDNA3.1‐based (HEK293T) or pSG5‐based (COS‐1) expression vectors, 300 ng firefly luciferase reporter (pM82) (Sterneck et al. 1992), 100 ng pLG4 renilla luciferase for normalization, and 2.5 μL (HEK293T) or 3 μL (COS‐1) Fugene HD. The mix was divided over three wells for triplicates. After 48 h, luciferase activity was measured using Dual‐Glo luciferase assay system (Promega) and detected with a GloMax‐Multi system (Promega).

### 4.5 Immunoblotting

Cells were washed in PBS and lysed in 50 mM Tris pH 8.0, 150 mM NaCl, 0.5% sodium deoxycholate, 0.1% SDS, 1% Triton‐X 100 with protease/phosphatase inhibitors (Roche). Lysates were sonicated, separated by SDS‐PAGE, and transferred to 0.2 μm PVDF membranes (Trans‐Blot Turbo, Bio‐Rad). Antibodies used: anti‐HA (MMS‐101R, Covance, 1:1000), anti‐β‐Actin (MP Biomedicals, 1:10,000), HRP‐anti‐mouse (GE Healthcare, 1:5000), and Lightning Plus ECL (Perkin Elmer). Membranes were reprobed with Restore Western Blot Stripping Buffer (Thermo Fisher) and detected using ImageQuant LAS 800 (GE Healthcare), and ImageQuant software was used to quantify the signals.

### 4.6 Generation of *NfCEBPA* ^*ΔuORF*^ Fish

Chemically modified guide crRNAs were synthesized by Integrated DNA Technologies (IDT). The crRNAs were designed using the IDT alt‐R guide RNA design tool, and three overlapping guide RNAs were selected (https://eu.idtdna.com/site/order/designtool/index/CRISPR_CUSTOM). A 105 bp oligonucleotide with 54‐ and 38‐bp homology arms, respectively, and two consecutive phosphorothioate modifications on both the 5′ and 3′ ends was synthesized by IDT to serve as the repair template. The target regions of the guide crRNAs and the repair template are listed in the table below. Injection mixes were prepared as per the protocol outlined at the IDT web site (https://sfvideo.blob.core.windows.net/sitefinity/docs/default‐source/user‐submitted‐method/crispr‐cas9‐rnp‐delivery‐zebrafish‐embryos‐j‐essnerc46b5a1532796e2eaa53ff00001c1b3c.pdf?sfvrsn=52123407_10). Briefly, crRNA and tracrRNA were diluted to 3 μM (1 μM for each crRNA) in IDT Nuclease‐Free duplex buffer and annealed by heating at 95°C for 5 min, followed by cooling to room temperature. The solution was mixed with an equal volume of 0.5 μg/μL Cas9 protein (alt‐R, IDT) in Cas9 working buffer (20 mM HEPES, 150 mM KCl, pH 7.5). Injections were performed in one‐cell stage eggs as described in (Valenzano et al. 2011). A total of 697 eggs were injected, with 100 animals surviving to adulthood. These were in‐crossed, and eggs from each breeding pair were collected and genotyped as described below. Three founders with the correctly edited base pairs were identified. The combined offspring of the 39 F1 animals were genotyped using tissues obtained by fin clipping. Four heterozygous males were identified and subsequently outcrossed twice.

**Table:** 

Guide crRNA 1	CCGGCTTTAGGTTCTCCATGGAG
Guide crRNA 2	CCTCGCCATGCCCGCCCGGCTTT
Guide crRNA 3	CCGCCCGGCTTTAGGTTCTCCAT
Repair template	TCCGACTCACCTGAGTGTGTGACTTTCCAAACTGGATTTCCGGAGCTCCTC GCCTTGCCCACCAGGTTTTAGGTTCTCCATGGAGCTCTCGAACCTGTACGA GGT

### 4.7 Genotyping

Samples (eggs/fin clips) were incubated in 50 μL 0.05 M NaOH at 95°C for 10 min, neutralized with 5 μL 1 M Tris–HCl (pH 8.0), and amplified for a 289‐bp region (Forward: 5′‐CTC TTC GTT CCA ACA CAA AGT G‐3′, Reverse: 5′‐GGT CTA TGG AGG TCT CGT TGT C‐3′). PCR products were digested with SexAI and analyzed by electrophoresis.

### 4.8 Fish Maintenance

Fish of the *Nothobranchius furzeri* strain ZMZ‐1001 were maintained at 28°C on a 12‐h light/12‐h dark cycle in 1.5‐ or 3.5‐L tanks connected to a recirculation water system (300 mL/min per individual tank), ensuring consistent water quality and temperature. The fish were fed frozen bloodworms (Chironomus, purchased from Dutch Select Food) twice daily on weekdays and once daily on weekends. At 6–7 weeks of age, fin clips were taken for genotyping. After genotyping, female fish with the same genotype were grouped into 3.5‐L tanks of four to five individuals, whereas male fish were kept separated in 1.5‐L tanks. For breeding, single heterozygous males and one to three heterozygous females, aged between 8 and 10 weeks, were placed together in a 3.5‐L tank containing a tray with autoclaved fine sand for egg collection. Eggs were collected from the sand trays by sieving and transferred to a Petri dish containing filter‐sterilized tank water. The eggs were then bleached by incubation for 10 min in sterile tank water containing 0.025% sodium hypochlorite, washed twice with sterile tank water, and transferred to a new Petri dish with sterile tank water containing gentamycin (10 μg/mL) and Methylene Blue (1:20,000), and incubated at 28°C. 48 h after the embryos reached diapause II, the eggs were transferred to Petri dishes containing autoclaved Whatman paper soaked in humic acid (1 g/L sterile tank water) and further incubated at 28°C until the embryos were fully developed. Hatching was induced by transferring the eggs to cold (4°C) humic acid (1 g/L sterile tank water) and incubating them at 28°C for 4 h. The hatched fish were placed together in tanks (max. 50 fish per tank). Feeding started the next day as described above. Two weeks after hatching, fish were transferred to individual tanks.

### 4.9 Lifespan Experiment

Wild‐type and homozygous *NfCEBPA* ^*∆uORF*^ mutants from heterozygous parents were included post‐genotyping (8–10 weeks). Lifespan was recorded from hatching until natural death or humane endpoint. Terminated fish were euthanized in ice water for 5 min. Dead fish were frozen at −20°C, fin‐clipped, and re‐genotyped. Survival differences were analyzed using Prism (GraphPad). The study was approved by the Dutch Central Commission of Animal Experiments (CCD, license no AVD1050020197765).

### 4.10 Aging Phenotypes

Body length was measured using a digital caliper for males that died between 293 and 321 days of age and females that died between 176 and 204 days. Fish health was assessed twice daily (once on weekends) by caretakers and twice weekly by scientists. Healthspan analysis recorded: visible tumors on the outside of the fish (outgrowth of cell mass or an increase in black pigmentation, excluding the tail fin); rapid loss of color; rectal prolapse; dropsy; weight loss (based on visible examination); abnormal swimming phenotypes, discriminating between belly sliding (fish struggle to lift from the bottom of the tank) for a duration of at least 7 days, floating (fish remain at the water surface), swimming upside down (belly facing upwards), unbalanced (position of the head is higher or lower than the tail), or sideways (fish swim tilted to one side at various angles).

### 4.11 RNA‐Seq Libraries, Differential Gene Expression and GO Terms Enrichment Analysis

Whole fish liver, brain, and skin and muscle samples were harvested from three *NfCEBPA* ^*ΔuORF*^ animals (aged 365, 367, and 408 days) and three wild‐type animals (aged 363, 367, and 373 days) from the lifespan cohorts, terminated at humane endpoint criteria. Total RNA was isolated using TRIzol Reagent (Invitrogen) according to the manufacturer's protocol. RNA‐seq libraries were prepared according to the Smart‐3SEQ protocol (https://doi.org/10.1101/gr.234807.118) using 40 ng of total RNA per sample. Libraries were sequenced using PE150 kit on an Illumina NovaSeq X Plus platform at Novogene (Munich, Germany) to an average depth of 6.8 million reads per library. Raw reads were processed with TrimGalore v.0.6.7 (https://doi.org/10.5281/zenodo.7598955) to remove adapters and polyA‐tails, DEuplicated using the rmdup command from seqkit v.2.3.0 (https://doi.org/10.1002/imt2.191) and mapped to the *N. furzeri* genome assembly Nfu_20140520 (NCBI accession GCF_001465895.1) using HISAT2 v.2.2.1 (https://doi.org/10.1038/s41587‐019‐0201‐4). Gene counts were calculated using featureCounts v.2.0.1 (https://doi.org/10.1093/bioinformatics/btt656). Genes with low expression were filtered out by retaining only those with counts per million (CPM) greater than 1 in at least two samples and then normalized using the upper‐quartile method. To account for potential unwanted technical variation, we applied the RUVs method from RUVseq v. 1.40 (https://doi.org/10.1038/nbt.2931) with *k* = 6 factors of unwanted variation, using all genes as negative controls. Biological replicates within each group were specified to guide the estimation of unwanted variation. The resulting factors were incorporated as covariates into the differential expression model. Differential gene expression analysis was performed using the edgeR package v.4.2.1 (https://doi.org/10.1093/nar/gkaf018) with TMM normalization. Unwanted variation factors were included in the generalized linear model (GLM). Pairwise comparisons between tissues of wild‐type and knock‐out strains were conducted using the exact test with a false discovery rate (FDR) threshold of 0.05. For GO term enrichment analysis, we used human homolog assignments for *N. furzeri* genes generated by Costa et al. (2025) (https://doi.org/10.1101/2025.01.28.63535). Enrichment analysis was performed using the clusterProfiler package v.4.12.6 (https://doi.org/10.1016/j.xinn.2021.100141), selecting the categories passing the Benjamini‐Hochberg FDR threshold of 0.05. Semantically similar terms were merged into one keeping the term with the lowest FDR value using the clusterProfiler simplify function (similarity cutoff = 0.5, similarity measure = Wang). Enrichment data were graphed as dot plots using the seaborn library (https://doi.org/10.5281/zenodo.883859) in Python v. 3.12. The plots were manually arranged into the final figure using Inkscape v. 1.4.2. KEGG pathway analysis was performed using functional annotation clustering analysis with the Data base for Annotation, Visualization, and Integrated Discovery (DAVID) and KEGG pathways as the only annotation category with medium stringency and an enrichment score > 2.5 using human homologs of the differentially regulated genes. Sequencing data were submitted to the European Nucleotide Archive under accession number E‐MTAB‐15325.

## Author Contributions

Study design: C.M., E.B. and C.F.C. Study conduct and data collection: C.M., J.S.M., K.U., G.K. and J.H. Data analysis and interpretation: C.M., J.S.M., K.U., E.B. and C.F.C. Manuscript writing: C.M., E.B. and C.F.C.

## Conflicts of Interest

The authors declare no conflicts of interest.

## Supporting information


**Figure S1:** acel70211‐sup‐0001‐FigureS1.pdf.

**Table S1:** *NfCEBPAΔuORF* vs. wild‐type differential regulated genes in liver, skin, muscle, and brain.

**Table S2:** acel70211‐sup‐0003‐TableS2.pdf.

## Data Availability

The data that support the findings of this study are openly available in European Nucleotide Archive (ENA) at https://www.ebi.ac.uk/ena, reference number E‐MTAB‐15325.
