# Supplementary material for: Enhanced C/EBPα Function Extends Healthspan and Lifespan in the African Turquoise Killifish
Source: Aging Cell. 2025 Sep 19;24(10):e70211. doi: 10.1111/acel.70211 (PMC12507421; doi:10.1111/acel.70211)
Supplement: Supplementary file 1 — Figure S1: acel70211‐sup‐0001‐FigureS1.pdf. [file ACEL-24-e70211-s003.pdf]

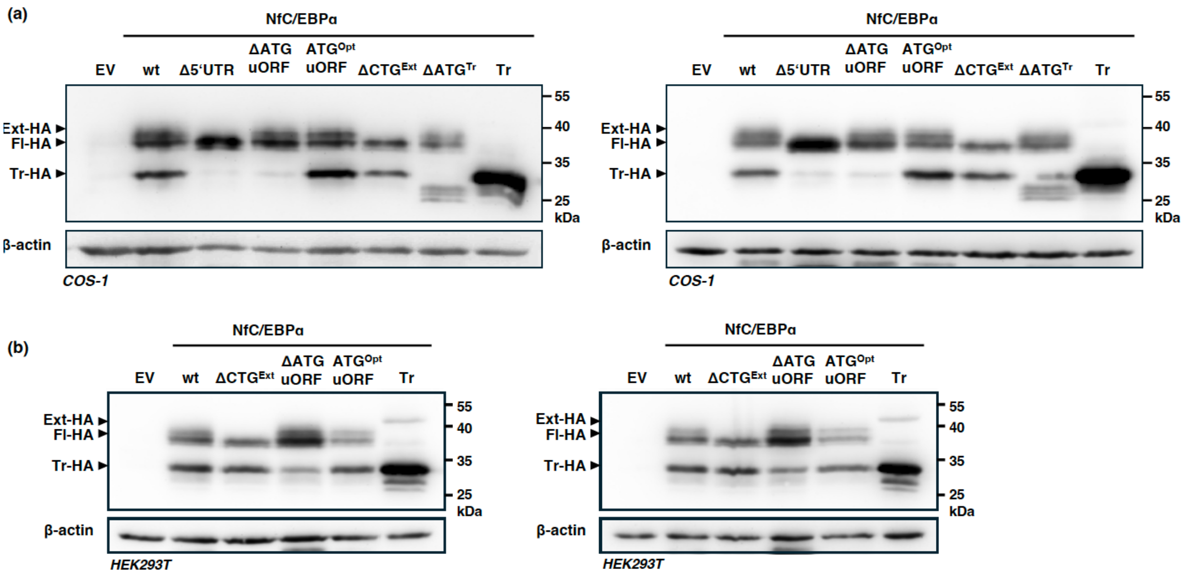

FIGURE S1

Differential translation of CEBPA mRNA into three NfC/EBPα protein isoforms. COS-1 (a) or HEK293T (b) cells were transfected with NfC/EBPα-HA expression vectors containing the following mutations: deletion of the entire 5'UTR (Δ5'UTR), deletion of the uORF-ATG (ΔATG), placing the uORF-ATG in a Kozak sequence for optimal translation initiation efficiency (ATG^{Opt}), deletion of the predicted extended-CUG initiation codon (ΔCTG^{Ext}), deletion of the double AUG predicted as an initiation site for the truncated isoform (ΔATG^{Tr}), and an expression vector for truncated (Tr)-NfC/EBPα only. β-actin served as a loading control.
