# Supplementary material for: Enhanced C/EBPα Function Extends Healthspan and Lifespan in the African Turquoise Killifish
Source: Aging Cell. 2025 Sep 19;24(10):e70211. doi: 10.1111/acel.70211 (PMC12507421; doi:10.1111/acel.70211)
Supplement: Supplementary file 3 — Table S2: acel70211‐sup‐0003‐TableS2.pdf. [file ACEL-24-e70211-s001.pdf]

Table S2. Go term enrichment analysis of *NfCEBPA*$^{\Delta uORF}$ vs. wild-type killifish.

Positive fold enrichment (red) means overrepresentation in *NfCEBPAΔuORF* , negative fold enrichment (blue) means underepresentation/depletion in *NfCEBPAΔuORF*

Organ	GO category	Contributing genes	Number of genes	Fold Enrichment	RAW p-value	FDR
Liver	GO:0043467 regulation of generation of precursor metabolites and energy	*atp7a, ddit4, gapdhs, grb10, hif1a, insr, irs2, ppara, ppif, ppp1r3g, psen1, rbpj, slc25a33, slc4a4*	14	5,68844697	1,5258E-07	0,000497432
	GO:0032868 response to insulin	*eef2k, errfi1, foxo1, grb10, hsd11b2, igfbp1, insig2, insr, irs2, lpin2, pck1, pid1, ppara, shc1, sik2, slc25a33, slc2a1, socs3, tns2, xbp1*	20	3,840301752	2,66648E-07	0,000497432
	GO:1903580 positive regulation of ATP metabolic process	*gapdhs, hif1a, insr, pid1, ppara, psen1, slc4a4*	7	10,98458725	2,35774E-06	0,002358887
	GO:1900544 positive regulation of purine nucleotide metabolic process	*gapdhs, hif1a, insr, pid1, ppara, psen1, slc4a4*	7	10,2759042	3,82567E-06	0,002378929
	GO:0071396 cellular response to lipid	*cpt1a, creb1, ddit4, errfi1, fgfr2, foxa1, foxo3, gnai1, gpr155, hdac6, hspa1b, insig2, klf9, lpl, nfkbia, pck1, pde4b, pid1, ppara, rxrg, sgk1, smarcd1, tgfb1, tnfaip3, tnip2, xbp1*	26	2,623496607	6,82519E-06	0,003092281
	GO:0006090 pyruvate metabolic process	*ddit4, gapdhs, hif1a, insr, pc, pck1, pfkl, pgam1, ppara, psen1, slc4a4*	11	4,767460317	1,87976E-05	0,004675585
	GO:0045913 positive regulation of carbohydrate metabolic process	*foxo1, gapdhs, hif1a, insr, irs2, ppara, ppp1r3g, psen1, slc4a4*	9	5,68844697	2,64723E-05	0,005487117
	GO:0051249 regulation of lymphocyte activation	*actl6a, arg2, bloc1s3, cd38, cebpb, foxo3, il6st, irs2, itpkb, junb, pck1, sdc4, slc7a1, smad7, smarcd1, tgfb1, tnfaip3, tnip2, xbp1, zbtb16*	20	2,645789288	7,27163E-05	0,006108204
	GO:0001773 myeloid dendritic cell activation	*clec4d, notch2, psen1, rbpj, tgfb1*	5	10,83513709	7,52245E-05	0,006108204
	GO:0072574 hepatocyte proliferation	*cebpb, hpn, notch2, tnfaip3, xbp1*	5	10,83513709	7,52245E-05	0,006108204
	GO:0002285 lymphocyte activation involved in immune response	*atp7a, clec4d, il6st, junb, notch2, pck1, plcl2, psen1, smad7, socs3, tgfb1, xbp1*	12	3,79229798	7,81302E-05	0,006108204
	GO:1902893 regulation of miRNA transcription	*foxa1, foxo3, hif1a, jun, notch2, ppara, tgfb1, wt1*	8	5,600932401	8,34946E-05	0,006108204
	GO:0048545 response to steroid hormone	*cd38, ddit4, errfi1, foxa1, foxo3, hdac6, hsd11b2, hspa1b, jun, junb, klf9, pck1, ppara, rxrg, sgk1, tgfb1, txnip*	17	2,865291807	9,75015E-05	0,006272036
	GO:0045598 regulation of fat cell differentiation	*cebpb, creb1, foxo1, hdac6, lpl, tfe3, tgfb1, trib2, xbp1, zbtb16*	10	4,17500695	0,000139585	0,007203993
	GO:0045637 regulation of myeloid cell differentiation	*cebpb, creb1, foxo3, hif1a, hspa1b, itpkb, mitf, nfkbia, notch2, tfe3, tgfb1, zbtb16*	12	3,523167155	0,000158017	0,007757381
	GO:1901654 response to ketone	*bcl2l1, cd38, creb1, ddit4, errfi1, foxo3, gnai1, hdac6, klf9, pck1, sgk1, tgfb1, txnip*	13	3,232778606	0,000200708	0,008273207
	GO:0050679 positive regulation of epithelial cell proliferation	*atp7a, fgfr2, hdac6, hif1a, hpn, irs2, lamb1, notch2, rbpj, tgfb1, tnfaip3, xbp1*	12	3,391869001	0,000225627	0,009051751
	GO:0070482 response to oxygen levels	*atp7a, cd38, cpt1a, ddit4, eef2k, egln3, fgfr2, foxo1, foxo3, hif1a, hsd11b2, pck1, plod1, ppara, slc2a1, tgfb1, tmbim6*	17	2,649413657	0,00024954	0,009535692
	GO:0007586 digestion	*asah2, cck, cd36, chia, enpp7, fabp2, pls1, si, slc5a1*	9	-11,04227941	1,12772E-07	0,000237046
	GO:0015669 gas transport	*hbg2, hbz, rhag, rhcg*	4	-33,65266106	4,59241E-06	0,00482662
	GO:0019755 one-carbon compound transport	*hbg2, hbz, rhag, rhcg*	4	-27,71395617	1,0707E-05	0,006424736
	GO:0050892 intestinal absorption	*cd36, enpp7, fabp2, pls1, slc5a1*	5	-16,35893246	1,22259E-05	0,006424736
Skin	GO:0009617 response to bacterium	*crp, cxcl10, mf213, tlr3, tlr6, usp18*	6	-12,10478589	4,87165E-06	0,003317595
	GO:0032729 positive regulation of type II interferon production	*cd226, cd276, tlr3*	3	-51,12340426	2,47056E-05	0,004178595
	GO:0002757 immune response-activating signaling pathway	*cd226, cd276, mog, tlr3, tlr6*	5	-12,02602603	3,79213E-05	0,004178595
	GO:0072676 lymphocyte migration	*cd69, cxcl10, saa1*	3	-32,91506849	9,2933E-05	0,004868257
Muscle	GO:0140966 piRNA-mediated heterochromatin formation	*ddx4, fkbp6, tdrd12*	3	-81,91363636	5,40265E-06	0,007606929
	GO:0141005 retrotransposon silencing by heterochromatin formation	*ddx4, fkbp6, tdrd12*	3	-63,01048951	1,27778E-05	0,00873943
	GO:0034587 piRNA processing	*ddx4, fkbp6, tdrd12*	3	-54,60909091	2,02244E-05	0,00873943
	GO:0031048 regulatory ncRNA-mediated heterochromatin formation	*ddx4, fkbp6, tdrd12*	3	-51,19602273	2,48279E-05	0,00873943
Brain	GO:0002088 lens development in camera-type eye	*adamts9, bfsp2, crybb1, crygb*	4	38,50641026	2,8201E-06	0,000667971
	GO:0007601 visual perception	*bfsp2, crybb1, crygb, kcnj10, rho*	5	19,25320513	4,04231E-06	0,000667971
